# Thinking process templates for constructing data stories with SCDNEY

**DOI:** 10.12688/f1000research.130623.2

**Published:** 2023-12-15

**Authors:** Yue Cao, Andy Tran, Hani Kim, Nick Robertson, Yingxin Lin, Marni Torkel, Pengyi Yang, Ellis Patrick, Shila Ghazanfar, Jean Yang

**Affiliations:** 1 Laboratory of Data Discovery for Health Limited (D24H), Science Park, Hong Kong SAR, China; 2 Sydney Precision Data Science Centre, The University of Sydney, Sydney, NSW, 2006, Australia; 3 Charles Perkins Centre, The University of Sydney, Sydney, NSW, 2006, Australia; 4 School of Mathematics and Statistics, The University of Sydney, Sydney, NSW, 2006, Australia; 5 Children's Medical Research Institute, The University of Sydney, Westmead, NSW, 2145, Australia

**Keywords:** single-cell analysis, data analysis, data story, thinking process template, living workshop

## Abstract

**Background:**

Globally, scientists now have the ability to generate a vast amount of high throughput biomedical data that carry critical information for important clinical and public health applications. This data revolution in biology is now creating a plethora of new single-cell datasets. Concurrently, there have been significant methodological advances in single-cell research. Integrating these two resources, creating tailor-made, efficient, and purpose-specific data analysis approaches can assist in accelerating scientific discovery.

**Methods:**

We developed a series of living workshops for building data stories, using Single-cell data integrative analysis (scdney). scdney is a wrapper package with a collection of single-cell analysis R packages incorporating data integration, cell type annotation, higher order testing and more.

**Results:**

Here, we illustrate two specific workshops. The first workshop examines how to characterise the identity and/or state of cells and the relationship between them, known as phenotyping. The second workshop focuses on extracting higher-order features from cells to predict disease progression.

**Conclusions:**

Through these workshops, we not only showcase current solutions, but also highlight critical thinking points. In particular, we highlight the Thinking Process Template that provides a structured framework for the decision-making process behind such single-cell analyses. Furthermore, our workshop will incorporate dynamic contributions from the community in a collaborative learning approach, thus the term ‘living’.

## Introduction

Recent advancements in biotechnology have empowered scientists to generate unprecedented amounts of data at the cellular level that carry critical information for important clinical and public health applications ( Goodwin, McPherson, and McCombie, 2016; Stark, Grzelak, and Hadfield, 2019). These data provide a unique opportunity for us to inspect individual cells through the lens of genomics, transcriptomics, proteomics and so on, providing insight into different aspects of a cell and representing a data revolution in biomedical data. To extract scientific discoveries from these data, over one thousand analytical methods have been developed ( Zappia and Theis, 2021) to exploit diverse kinds of data and answer a broad range of questions. These analytical methods can be used as ‘black box’ tools to analyse data without knowledge of the methodological details. Hence, it can be difficult to determine how ‘robust’ a data analysis should be conducted. Hence, this can create challenges when it comes to deciding how rigorous a data analysis is. To make the most of the single-cell data revolution in omics science, it is important for researchers to first navigate and determine the optimal analytical tools for each question while being aware of their hidden pitfalls and assumptions.

Analysing omics data often involves complex workflows including data cleaning, processing, and downstream analysis. A critical component in a successful analysis is the thinking process, which involves the analyst considering the steps in the workflows and making informed decisions that are appropriate for the research questions at hand. For example, the workflow for single-cell analysis often involves multiple interdependent steps such as data filtering and normalisation, feature selection, clustering, dimensionality reduction, alongside further downstream analytical steps. Each of these steps can require analysts to make context-specific decisions, such as deciding thresholds (e.g., filtering or feature selection), selecting parameters (e.g., normalisation or clustering) or selecting an algorithm (e.g., dimensionality reduction). As these analytical choices are dependent on earlier steps, they can have cascading impacts on the downstream analysis, and eventually, the conclusions that are drawn ( Krzak *et al.,* 2019; Raimundo, Vallot, and Vert, 2020). Workflows can help users identify a set of seemingly disjoint methods into a unified and coherent process. Thus, it is crucial that users are guided through the thinking process in order to make the most appropriate decisions at each step given their specific context.

There is a difference between offering a tutorial or workflow and offering a thinking process. Computational methods are often accompanied by a tutorial that demonstrates how to apply the method to perform a specific task on an example dataset. These tutorials can be straightforward to follow and understand, helping users run the method on their own data. Workflows describe a sequence of analytical methods for processing and analysing certain types of data ( Breckels *et al.,* 2016; Lun, McCarthy, and Marioni, 2016; Borcherding, Bormann, and Kraus, 2020). Workflows can help users identify a set of seemingly disparate methods into a cohesive whole. However, simply copying an existing tutorial or workflow leaves the risk of treating the methods as a ‘black box’, potentially leading to false discoveries. We believe that it is important to not only instruct analysts on how to apply a method or workflow, but also to guide them to critically assess their results at each stage. Indeed, efforts are underway to make more transparent what happens ‘behind the paper’ such as the Springer Nature protocols and methods community ( https://protocolsmethods.springernature.com/channels/behind-the-paper) with discussions surrounding experimental and analytical choices throughout the project. Critical thinking and assessment of results at each stage enables the analysts to identify where problems arise and guides them to customise their analysis for their specific context. Thus, there is a pressing need to build on existing tutorials and workflows in a way that incorporates such critical thinking.

To this end, we present a Thinking Process Template to formalise the thought process an analyst should undertake to ensure robust analysis that is tailored to their data. We use Thinking Process Template as a general term to refer to the data analysis procedure from data input to final analysis outcome that involves critical thinking processes and goes beyond the simple application of tools or the products of data analysis. Here, we demonstrate this through scdney, a collection of analytical packages and living workshop materials, which can be updated based on feedback and suggestions from users. In this paper, we demonstrate two examples of our Thinking Process Template in inferring and assessing a cell lineage trajectory, and in performing patient disease classification. We envision that our Thinking Process Template and scdney’s living workshops will complement existing resources and will be a model for future tutorials to encourage transparent and robust research practices for the bioinformatics and biomedical data science community.

## Methods

### Selection of data stories to illustrate scdney

Here we showcase two data stories to illustrate scdney. These two data stories and the accompanying workshops were not derived from previous studies nor have they been published elsewhere. The first data story describes the use of scdney on cell level analysis through inferring and assessing the developmental trajectory of individual cells. The second data story details the use of scdney on patient level analysis by extracting and summarising information obtained from each cell. The code for both data stories are hosted on Github as reproducible Rmarkdown files, reported in the code availability section. The underlying data are reported in the data availability section.

### Workshop for data story 1

A summary of the case study is provided below with detailed information including R code hosted on our Github ( Lin, Kim and Chen, 2023).

To predict the gene pairs associated with the developmental course of the differentiation of mouse hippocampal cells, we downloaded the publicly available data (from GEO with accession number GSE104323) profiling eight cell types from neural lineages of the mouse hippocampus harvested from two post-natal timepoints (day 0 and 5) ( La Manno *et al.,* 2018). For speed, we removed the Nbl1, Nbl2, Granule and CA cell types from the dataset and reduced the dataset from 18,213 to 12,935 cells. To evaluate the accuracy of the original cell type labels, we applied scReClassify ( Kim *et al.*, 2019) from the scdney package. scReClassify generates cell-type-specific probabilities for each cell, where a probability of 1 denotes the highest accuracy in classification and 0 denotes lowest accuracy. Using the maximum probability assigned to each cell, we re-labelled the cell-type annotations of cells that have inconsistent labels and have a maximum probability greater than 0.9. Then, we used the re-labelled cell-type annotations to perform marker gene analysis using Cepo, a method to determine cell-type-specific differentially stable genes ( Kim *et al.,* 2021).

To build the trajectories, we applied two commonly used trajectory inference tools, Slingshot ( Street *et al.,* 2018) and destiny ( Angerer *et al.,* 2016). Finally, to predict gene-pairs that change over the trajectory course, we used our previously developed package scHOT ( Ghazanfar *et al.,* 2020), which is available on Bioconductor. scHOT enables detection of changes in higher-order interactions in single-cell gene expression data.

### Workshop for data story 2

A summary of the case study is provided below with detailed information including R code hosted on our Github ( Cao and Tran, 2023).

We predict patient disease outcome using COVID-19 datasets and the packages scFeatures ( Cao *et al.,* 2022), and ClassifyR ( Strbenac *et al.,* 2015). To build the prediction model on distinguishing mild and severe outcomes, we used the publicly available Schulte-Schrepping data ( Schulte-Schrepping *et al.,* 2020). We randomly sampled 20 mild and 20 severe patient samples for the purpose of demonstrating the workshop in a reasonable amount of time. Then, we applied scFeatures from the scdney package to generate patient representations from the single-cell data. scFeatures generates interpretable molecular representations from various feature types. By doing so, we were able to represent each patient with more information than a matrix of gene expressions. At the same time, it also transformed the scRNA-seq data into a matrix of samples by features, which is a standard form for machine learning models. We generated a total of 13 matrices, one for each feature type across the feature categories of (i) cell type proportions, (ii) cell type specific gene expressions, (iii) cell type specific pathway expressions, (iv) cell type specific CCI scores and (v) overall aggregated gene expressions. The details of the feature types can be found in the scFeatures publication ( Cao *et al.,* 2022).

To build a patient outcome classification model from the patient representations, we used our previously developed package ClassifyR ( Strbenac *et al.,* 2015), which is available on Bioconductor ( https://bioconductor.org/packages/ClassifyR/). ClassifyR provides an implementation of cross-validated classifications, including implementation for a range of commonly used classifiers and evaluation metrics. For this case study, we ran SVM on each of the feature types using a repeated five-fold cross-validation framework with 20 repeats. The accuracy was measured using the metric ‘balanced accuracy’ that is implemented in ClassifyR.

To assess the generalisability of the constructed model, we used the Schulte-Schrepping data as training data and another publicly available COVID-19 scRNA-seq dataset, the Wilk data ( Wilk *et al.,* 2020), as an independent testing data. First, we processed the dataset in the same way using scFeatures to generate the patient representations. Given that different datasets generate slightly different sets of features, for example, due to the difference in the genes recorded, we subset the features derived from the Schulte-Schrepping dataset and the Wilk data by their common features. We then rebuilt the model using the Schulte-Schrepping dataset as the training dataset using the same cross-validation framework as above. The best model from the 100 models (i.e., from the 20 repeated five-fold cross-validation) was identified based on balanced accuracy and evaluated on the Wilk dataset.

## Results

### Thinking Process Template

**Figure 1.**
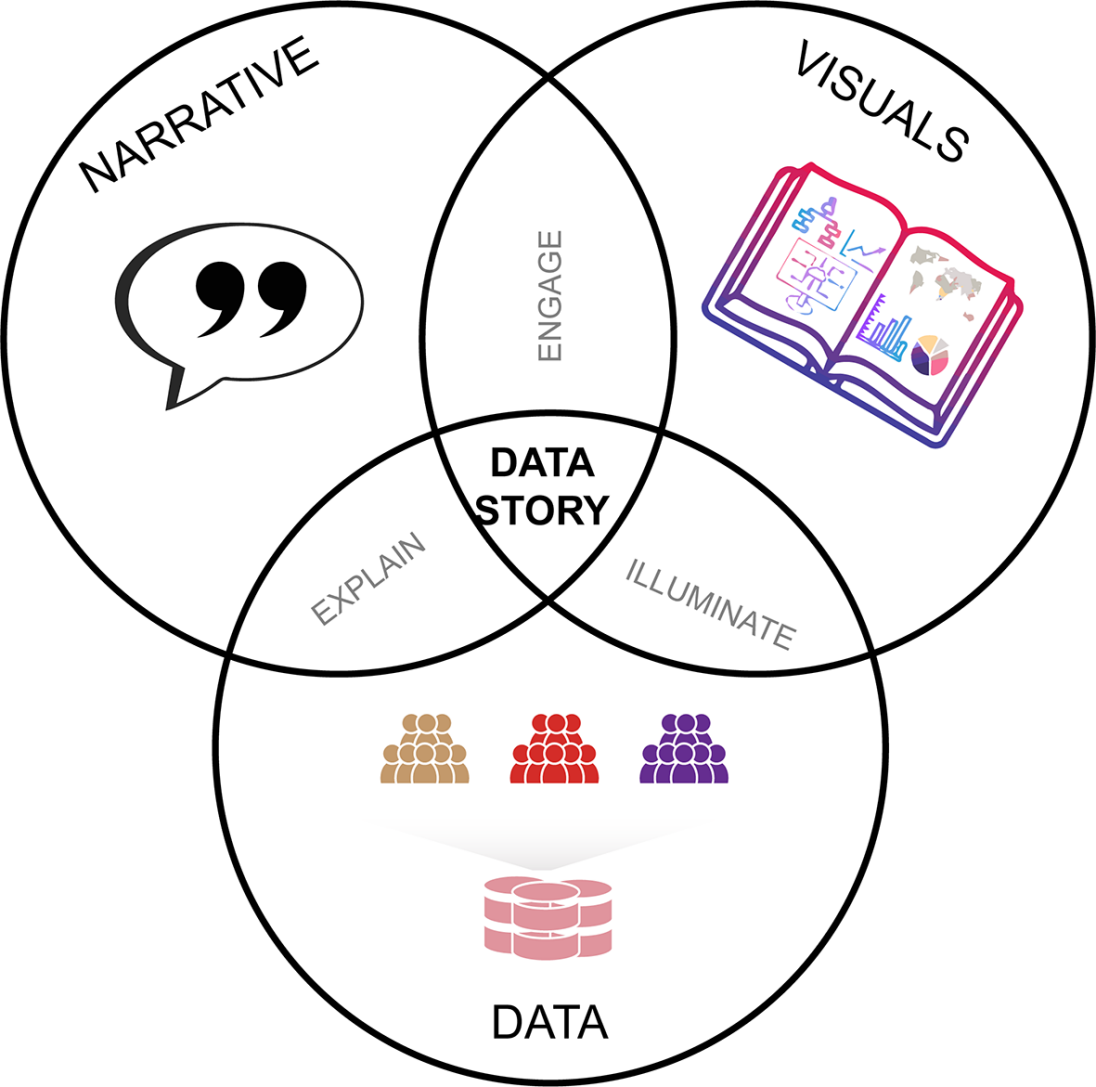
Critical thinking questions from the data drive our decision with the Narrative and illuminate with the Visuals.

Typically, in scientific research papers involving cellular data technologies, there are three key components: (1) Data, (2) Narratives, and (3) Visuals ( Figure 1). Through narratives, we explain the data; through visuals, we illuminate the data; through narratives and visuals we engage. At the intersection of the three components are the product: the data stories. However, what is hidden behind these components are the critical thinking questions such as evaluation and parameter choices that happen behind the decision-making process.

Here, we present a Thinking Process Template, to uncover the thinking process behind the construction of data stories, guided by analytical decisions. We demonstrate this in two distinct data analytical scenarios, presented as scientific questions. First, we ask, what are the cell types present in our developmental single-cell dataset, and what are the correlated gene pairs in each trajectory? Second, what features are important for disease outcome classification? In both cases we illuminate the underlying thinking strategy taken by analysts/data scientists in extracting biological knowledge from the data and drawing from the vast compendium of prior knowledge to reveal novel scientific knowledge.

*Scdney - Single cell data integrative analysis*

**Figure 2.**
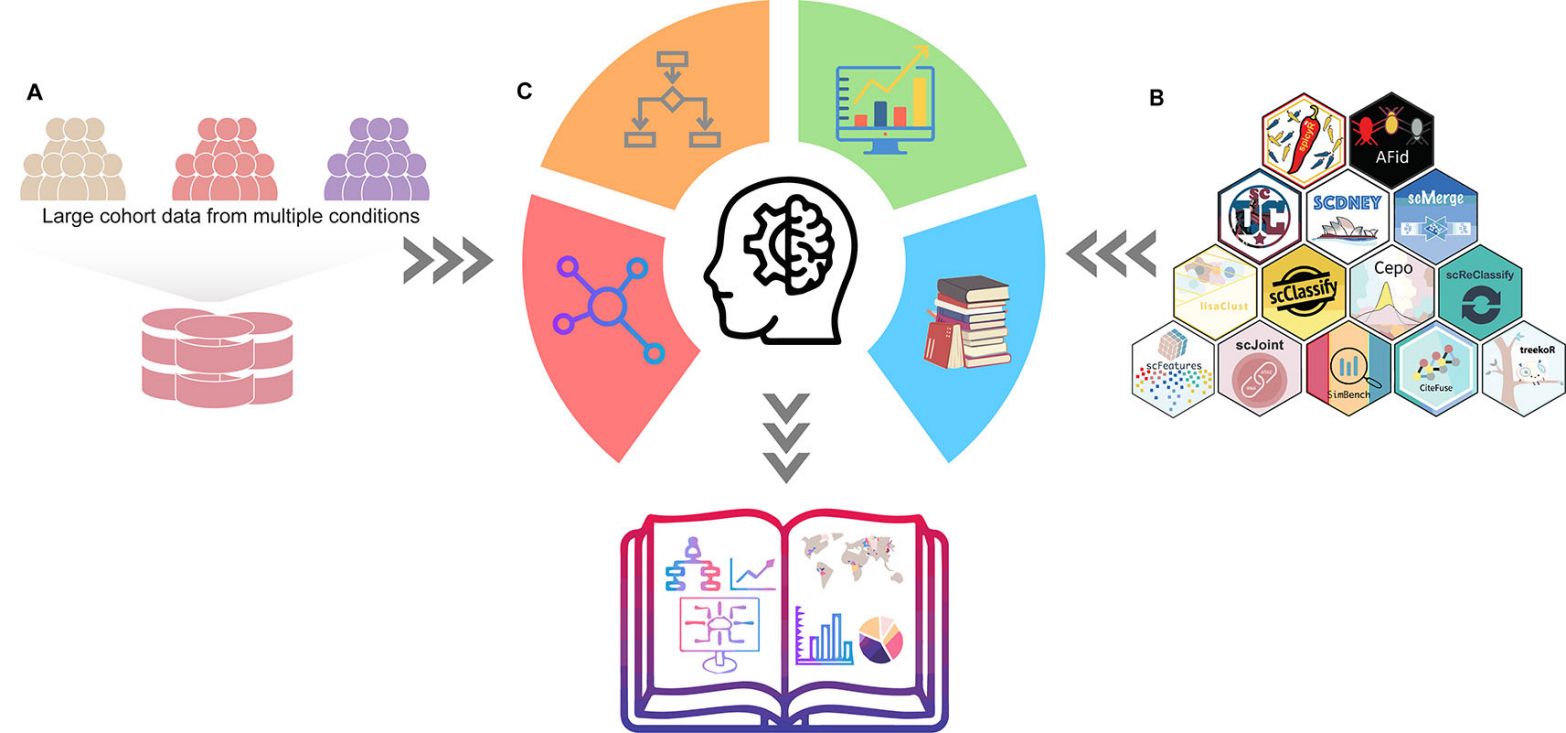
scdney workflow. (A) *Collection of Data* - The data stories start with data. (B) *Collection of methods of scdney* - The collection of methods are used for the computational analysis of data. (C) *Critical Thinking* - Through critical thinking, we derive the final data story.

As a vehicle to demonstrate the Thinking Process Template, we present scdney ( Figure 2), a series of foundational methods for single cell data analysis, including

• a data integration approach for scRNA-seq data that enables tailored prior knowledge ( Lin *et al.,* 2019);
• a novel cell type classification method based on cell hierarchy ( Lin *et al.*, 2020);
• a novel method for identifying differential stable genes, that is, genes that are stably expressed in one cell type relative to other cell types ( Kim *et al.,* 2021);
• a multi-modal workflow for analysing CITE-seq data ( Kim *et al.,* 2020);
• an analytical approach to test for higher-order changes in gene behaviour within human tissue ( Ghazanfar *et al.,* 2020). By higher-order changes, we refer to higher order interactions such as variation and coexpression that are beyond changes in mean expression; and
• a feature extraction method that creates multi-view feature representations on patient level from single-cell data ( Cao *et al.,* 2022).

Building upon the collection of vignettes, the Thinking Process Template examines various critical thinking questions that analysts need to make, which drives the decision for the next step in the analysis workflow. Next, using the scdney workflow, we will illustrate the process of generating two data stories. The scdney workflow starts with data ( Figure 2A), the series of methods are used for the analysis of data ( Figure 2B), and through the critical thinking ( Figure 2C), we derive the final data story.

*Narrative for data story 1 - to identify key gene-pairs associated with the developmental course*

**Figure 3.**
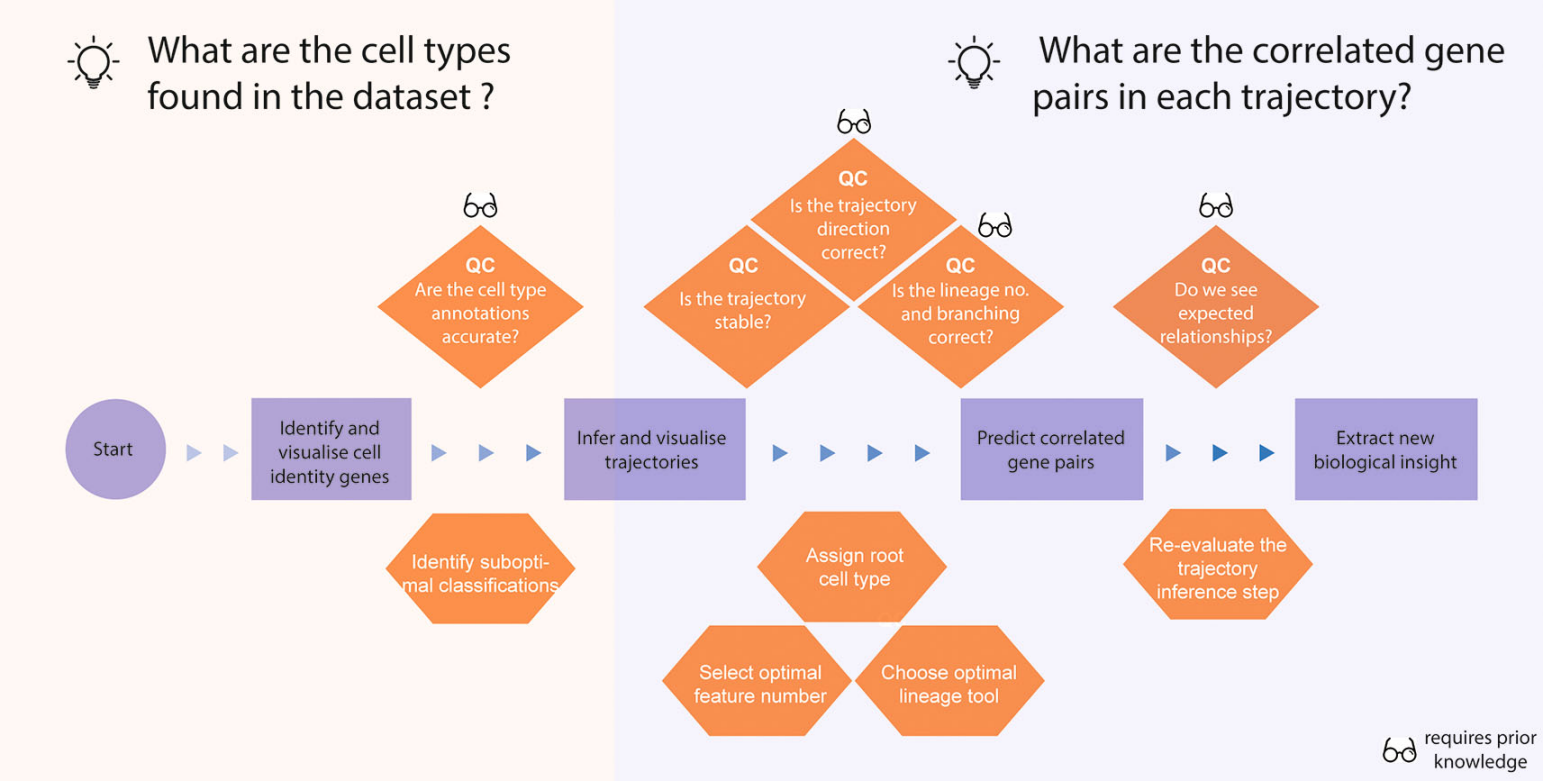
Thinking process template for analysing a single-cell RNA-seq data with a lineage trajectory. The thinking process begins from the processed data with cell type annotations and proceeds to constructing a trajectory and extracting biological insight through identification of correlated gene pairs. The orange diamonds highlight potential questions that help us quality check the data analysis, and the orange hexagonal shapes denote the specific computational tasks that are required to answer the questions above.

In the first data story, the aim was to identify key gene-pairs associated with the developmental course of the differentiation of mouse hippocampal cells, enabling us to find key gene sets that distinguish hippocampal development in mice using scRNA-seq data ( La Manno *et al.,* 2018) ( Figure 3). Box 1 lists some questions and our thought process during the development of the story.

Box 1. **Critical questions to consider for identification of key gene sets in the developmental course.**

**Question: Which tools should I use and what format does the data need to be in?**

**Thinking process:** Several tools have been developed to construct trajectories from single cell data. Different tools may require different types of input data; therefore, it is important to understand the tools and your data before selecting a tool. Another key aspect of working on trajectory reconstruction is to judge which cell populations to include in the trajectory analyses. Some cell types or cell populations not involved in the differentiation system of interest should be excluded in the trajectory inference.

**Question: Which trajectory method should I use?**

**Thinking process:** Depending on the complexity of the trajectory, the choice of tools can have a large impact on the accuracy of the resulting trajectory built. A large body of work has been performed to evaluate current single-cell trajectory inference methods ( Saelens *et al.*, 2019). They provide guidelines and a framework to test which trajectory tool and setting are most appropriate for your data. Again, this requires you to have a good understanding of the expected underlying biology in your data, such as the topology and the number of branches of the expected trajectory.

**Question: Are the cell type labels accurate?**

**Thinking process:** Evaluating the quality of the cell type labels is important, as the quality of this may directly impact downstream analyses such as determining cell-type markers. By quantifying the proportion of cells accurately labelled in the dataset, we are not only able to assess the quality of the overall dataset, but also to re-classify any mislabelled cells.

**Question: Is the trajectory stable?**

**Thinking process:** This can be achieved in many ways, such as testing the reproducibility of the trajectory when different tools are used or when permuting the features (gene sets or cells) in the data. A consistent trajectory across various permutations provides stronger support for the final trajectory.

**Question: Is the trajectory sensible?**

**Thinking process:** Inspecting how sensible a trajectory is critical. We should inspect various features of the trajectory such as the direction of the trajectory (which includes evaluating the root of the trajectory), the number of branches, and the number of terminal nodes (e.g., terminal populations) in the data. Whilst these evaluations require an in-depth understanding of your biological system through literature search, there are computational tools that help guide this. For example, CYTOTRACE can be used to predict the root cell (i.e., the most undifferentiated cell) in a cell population.

**Question: How reliable are the top regulated gene-pairs?**

**Thinking process:** This question essentially asks whether the extracted gene-pairs are expected for the current biological system. This often requires prior knowledge of experimentally validated ground truths, which can be employed to evaluate the validity of our results. The presence of one or more biological truths increases the confidence that the current framework is appropriate.

**Question: How accurate are the identified top gene-pairs?**

**Thinking process:** It is important to bear in mind that the presence of known biological truths in our results do not necessarily mean that the other predicted gene pairs are also biological truths. There are many ways we can validate the accuracy of the predicted gene-pairs, and these validation approaches can be done experimentally or computationally. Computationally, one of the ways we can validate the accuracy is to assess the reproducibility of our framework on a new dataset derived from the same biological system. When such independent datasets are not available, a simple train-test split can be performed on the data to test the reproducibility of the findings.

The dataset we use contains eight cell types from neural lineages of the mouse hippocampus harvested from two post-natal timepoints (day 0 and 5) ( La Manno *et al.,* 2018). Whilst the main goal in the original study was to demonstrate the RNA velocity fields that describe the fate decisions governing mouse hippocampal development, our data story aims to uncover novel gene-pairs associated with these neural lineages using scHOT ( Ghazanfar *et al.,* 2020).

**Figure 4.**
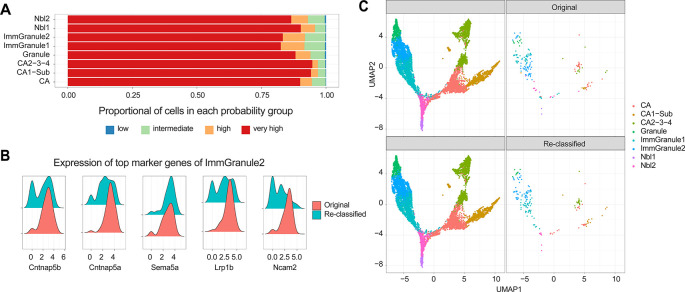
Assessment of cell type labels and re-annotation of sub-optimal labels with scReClassify. (A) Shows the proportion of cells in each confidence level, defined by scReClassify, for each cell type group. (B) The distribution of gene expression of top five marker genes in Immature Granule 2 cells as per the original labels (bottom panel) and re-classified labels (top panel). (C) UMAP of mouse brain cells coloured by cell type and faceted by cells that maintain their original labels (left) and those that have been re-classified (right).

We start by asking whether the cell type annotations in the original data are accurate. Here our expectation is that most of the labels are accurate, and by using scReClassify ( Kim *et al.,* 2019) we demonstrate that approximately 88.4% of cells show an original classification accuracy over 0.9 ( Figure 4A). Among these cells, only 1.5% (177 cells) were re-classified, suggesting that a small proportion of cells may have been mislabelled. These findings were confirmed through marker analysis using Cepo ( Figure 4B), and the cells with high confidence scores were re-labelled for subsequent analyses. Once we have confirmed with further quality control questions as shown in the box and ensured the quality of the cell type annotations, we can then use these labels to perform marker gene analysis and to construct the lineage trajectories ( Figure 4C).

After performing the quality control of the original annotations, we then can ask questions relating to trajectory reconstruction. In the trajectory building stage, we ask questions (see Box 1) to ensure the stability and robustness of the trajectories by testing the concordance of the pseudo-times between various trajectory reconstruction tools ( Cao *et al.*, 2019; Street *et al*., 2018). In our Thinking Process Template, we indicate at various points at which one can use prior knowledge (indicated by the glasses icon) to guide the analysis. For example, we can use prior knowledge to ask whether the reconstructed trajectories show the correct branching expected in the underlying biology of the differentiation and whether key gene-pairs that are known to be co-regulated are identified by scHOT ( Ghazanfar *et al.*, 2020), as well as asking which genes are differentially expressed across a pseudotime using tradeSeq ( Van den Berge *et al.*, 2020) and performing functional annotation of these gene sets through clusterProfiler ( Yu *et al.*, 2012). Together, these analyses demonstrate that the final trajectories are in line with our expectations and provide more confidence in the new biological insights extracted from these trajectories. The story includes other downstream analysis of the data such as cell-cell communication using CellChat ( Jin *et al.*, 2021) and RNA velocity analysis using scVelo ( Bergen *et al.*, 2020), which the users can perform to further explore their data.

*Narrative for data story 2 - develop a PBMC biomarker model to predict COVID-19 patient outcomes*

**Figure 5.**
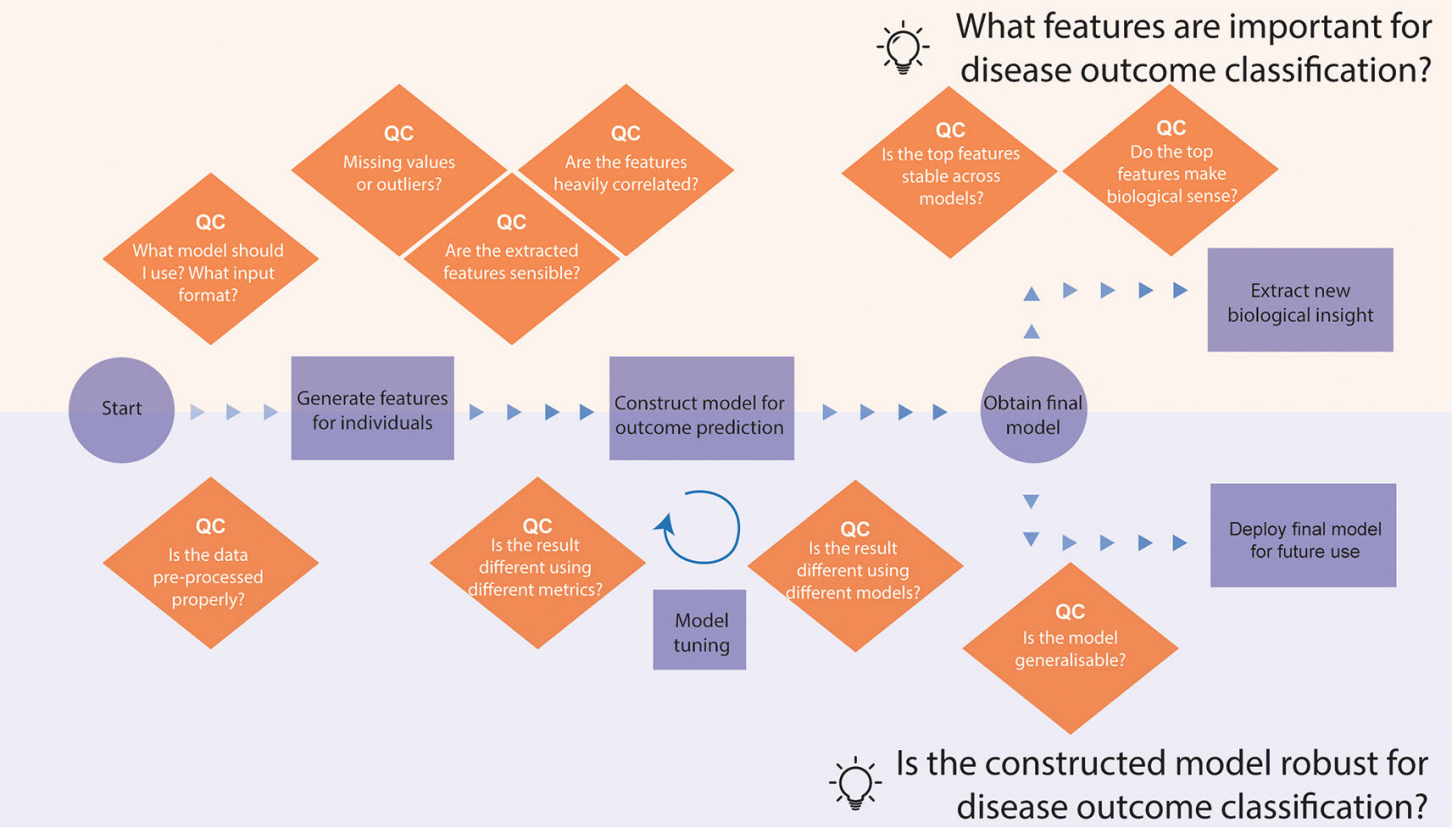
Thinking process template for analysing a single-cell RNA-seq data for disease outcome classification. The thinking process begins from processed data with cell type annotations and branches into two questions, each with a different focus. The top part focuses on using the disease classification model to extract biological insights into the disease, such as what features are important towards disease classification. The bottom part focuses on examining the model properties, such as whether the model is generalisable.

In our second data story, we aim to predict COVID-19 patient outcomes (mild or severe) from scRNA-seq data of peripheral blood mononuclear cells ( Schulte-Schrepping *et al.,* 2020) ( Figure 5). Below, we list some questions and our thought process during the development of the story. Here, we showcase the story we derived on the COVID-19 patient outcome prediction. The story begins with the question of what models and input format we will use to build a prediction model (see Box 2). Here, we decided to use classical machine learning instead of deep learning, given the small sample size of 20 mild and 20 severe patients. We utilise scFeatures, a package that generates interpretable multiscale features from scRNA-seq data, such as cell-type proportions, pathway expression, ligand-receptor interactions and more. These features can then be used as input to facilitate an interpretable classification model. Once we have asked quality control questions as shown in the box and ensured the quality of generated features, we then used these features to build models to predict mild or severe outcomes.

Box 2. **Critical questions to consider for the prediction of patient outcomes.**

**Question: What model should I use and what data structure is required by the model?**

**Thinking process:** There exist a number of advanced deep learning tools that can obtain various biological insights from the count matrix ( Bao *et al.*, 2022). However, a small sample size, which is often what’s typical in single-cell patient data, may not be ideal to train a deep learning model. We might consider the alternatives such as classification machine learning methods like random forest. These methods requires the input in the format of samples by features. In this case, we can consider manually extracting the features such as cell type proportion.

**Question: Is the data preprocessed appropriately?**

**Thinking process:** The quality of the data itself has a direct impact on the quality of the extracted features, and subsequently the quality of the model. Therefore, it is important to perform “quality control” both on the original count matrix and on any of the extracted features derived from the count matrix.

**Question: Should I downweight any samples?**

**Thinking process:** Class imbalance can have a negative effect on the model, as the model would be biassed towards the over-represented class. One potential strategy to alleviate this is to downweight the over-represented class.

**Questions: Do the generated features make sense? Are the extracted features sensible?**

**Thinking process:** This is really asking whether the extracted features are expected. This often requires finding a handful of the top differentially expressed genes through DE analysis and checking if they are mentioned in literature.

**Question: Does the overall graphical representation of the features look sensible?**

**Thinking process:** In this question, we are looking at the overall distribution of the generated features. For example, if we examine the heatmap or volcano plot, are we seeing what we expect to see? Also, see below for examples of quality control checks.

**Question: Are there any missing values or outliers in the generated features?**

**Thinking process:** We should inspect the generated features to ensure they are not saturated with missing values. Features where many values are missing may not be informative for downstream analysis and should be removed prior to model building.

**Question: Are the generated features heavily correlated?**

**Thinking process:** Having many heavily correlated features can negatively affect a model by introducing noise and instability.

**Question: There are a lot of the generated features, how do I make sense of them?**

**Thinking process:** Given the number of features in a single-cell matrix (typically around 20,000 genes for a scRNA-seq data), one may end up with many derived features. One strategy is to perform an association study, where we examine the association of the features with the outcome. We could also conduct a literature search or consult with biologists to determine whether these top features are biologically significant.

**Questions: How good is my prediction? How does it compare to the current state-of-the-art?**

**Thinking process:** The expected accuracy of a prediction can vary depending on the specific task at hand. For example, an accuracy of 0.6 may be what the current state-of-the-art is for a difficult disease classification task, whereas for a clear cell type classification task, an accuracy of 0.9 may be the baseline.

**Questions: Is the result different using different metrics? Different models?**

**Thinking process:** It may be necessary to try a number of machine learning models and a number of evaluation criteria to assess model performance. For example, when there are imbalanced class sizes, balanced accuracy and F1 score are better measures of model performance compared to precision and recall.

**Questions: Is my model overfitting to the data? Do I need further testing?**

**Thinking process:** One needs to be careful with model overfitting. A model may have very high accuracy on the dataset it is built from, but performs poorly on an unseen dataset. To assess model overfitting, we could test the performance of the model on an unseen dataset to assess its generalisability.

**Question: Are the top features stable across the models?**

**Thinking process:** After we obtain the model, we may wish to inspect the top features selected by the model. The repeated cross-validation framework is often used when building machine learning models as it provides a better assessment of model predictability than a simple train-test split. Therefore, we need to check whether the top features are similar across all models from the cross-validation framework.

**Figure 6.**
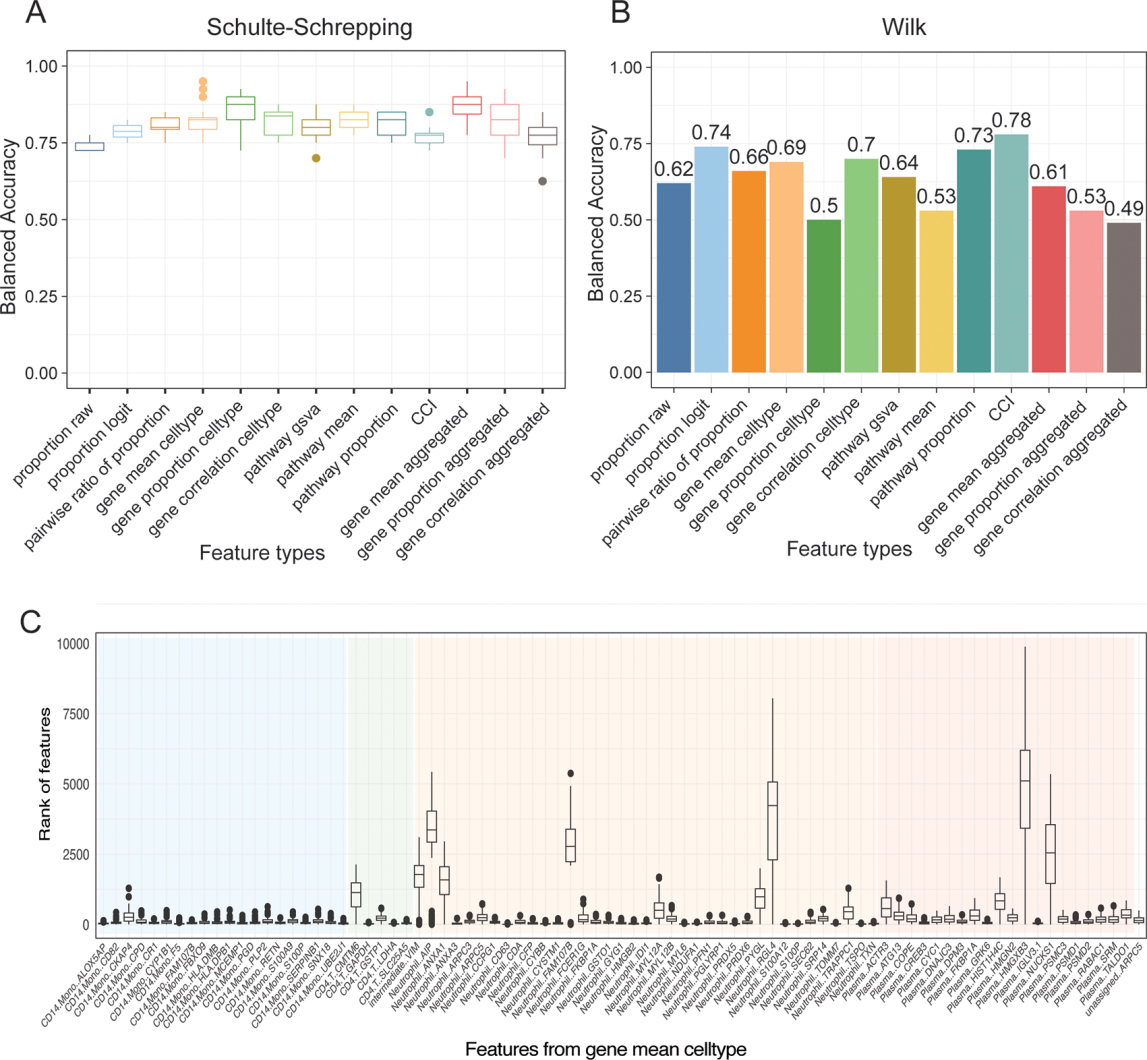
Assessment of disease outcome classification accuracy using scFeatures' generated features. (A) Shows the balanced accuracy of each feature type on classifying the mild and severe patients in the Schulte-Schrepping dataset. Models were run using five fold cross-validation with 20 repeats. For each feature type, the best model from the cross-validation was then selected and used to predict on the mild and severe patients in the Wilk dataset, as shown in (B). (C) Rankings of each feature in the feature type “gene mean celltype” across all cross-validated models.

When building machine learning models, it is crucial that we ask questions on the model performance on a variety of models and metrics. Therefore, we choose to use ClassifyR, as it provides a user-friendly implementation on a number of common machine learning models and evaluation metrics. We created models for each feature type, resulting in a total of 13 models and compared the utility of these feature types for patient classification. We found that a support vector machine classifier consistently achieves a cross-validation accuracy over 0.7 ( Figure 6A), demonstrating the usefulness of these features to classify disease outcomes.

Once the final models are obtained, we ask questions on the robustness of the models. One approach on this involves assessing the generalisability of the model on an independent dataset. We examined the performance of the 13 models on a different COVID-19 dataset obtained from the Wilk study that also contain mild and severe patients ( Wilk *et al.,* 2020). We found while the 13 models have close balanced accuracy between 0.75 and 0.88 on the Schulte-Schrepping dataset, their performance varied greatly in the Wilk dataset and ranged between 0.49 to 0.78 ( Figure 6A, B). It is noteworthy that models built from feature types such as “gene proportion cell type” that have high accuracy do not necessarily maintain good performance on the Wilk dataset. On the other hand, the feature type “CCI” achieved an accuracy of over 0.75 in both datasets, indicating potential for further examination. Finally, to extract biological insights from the fitted models, we guide users to interpret the fitted models to identify important features and reflect on whether the features make sense. Here, it is important not to select the top features based on a single model, but to ask about the stability of these top features. To illustrate this idea, we examined all features that appeared at least once as top 10 features in the cross-validated models. Figure 6C highlights that while the majority of the features were consistently ranked as top features across all models, a proportion of features were ranked in the hundreds and thousands position in some models. These two scenarios illustrate the importance of critical thinking to avoid heading down a wrong decision path.

## Discussion

Here, we have presented a Thinking Process Template to not only guide users how to perform a single cell data analysis, but also to encourage critical thinking, ensuring that each part of the workflow successfully performs its desired task. We demonstrated this through the use of scdney, a collection of analytical packages that can perform a wide range of single-cell data processing and analyses. In the previous section, we demonstrated the importance of the process with two examples: identification of key gene pairs that distinguish hippocampal development in mouse cells and generation of features from human cells for disease outcome prediction. We envisage use of the Thinking Process Template as a valuable framework for critical thinking in single-cell data analysis.

Bioinformatics analysis workflows involve many steps, each often requiring decisions to be made, dependent on the earlier choices. The most appropriate decisions will differ between datasets and analyses. Therefore, performing a robust analysis requires significant training and experience. However, our Thinking Process Template conveys this training as critical thinking questions that less-experienced users can easily follow for their specific context. The template can be adapted to a wide range of analyses, complementing the existing learning resources, to lower the barrier to entry for performing reproducible bioinformatics analysis. Furthermore, the template enables an asynchronous learning approach ( Bishop and Verleger, 2013), where the users can learn at their own pace and on their own time without the constraints of traditional workshop schedules. This is particularly useful for bioinformatics analysis, where the decisions and steps can vary depending on the specific datasets and analyses and need to be thoroughly thought about prior to drawing conclusions.

In the last decade, partly in response to the replicability crisis ( Guttinger and Love, 2019), there has been an increased emphasis on open and transparent science and an increased culture among bioinformaticians of sharing data and code so that key findings can be reproduced. However, sharing code alone does not address all aspects of the replicability of scientific conclusions and further, does not explicitly contribute towards the sharing of analytical strategies. In our Thinking Process Template, we believe acknowledging the critical thinking steps ensures a better understanding of the stability and robustness of analytical decisions made in an analysis, making it possible to assess if the same conclusions would be drawn if different decisions were made. Further, sharing the key critical thinking steps of a project, in addition to the code, will improve replicability of results by making it clear where, when, and why analyses can differ when the same code is applied to different data. This will enhance reproducibility of studies performed by different researchers and institutes, and by promoting open examination of the practices, may help to promote replicability in the broader research field.

The thinking process of data analysis is dynamic, constantly evolving and specific to the dataset and the research questions. In practice, when addressing similar research questions, the data analysis workflow that works well on one dataset may not be universal to all other datasets. The thinking process proposed in this paper could serve as useful tips and tricks to address these problems. The output from the thinking process can potentially stimulate a new thinking process, which may further inspire the scientists to ask different questions about the data. The complex thinking process involved in publication is starting to be acknowledged on collaborative learning platforms, such as the one established by F1000. These platforms enable authors to describe the behind-the-scenes stories leading to their publications, as well as for others to contribute analytical suggestions and ideas in a dynamic way. It is known that groups of people with cognitive diversity are often able to solve problems more effectively than a group of cognitively similar people ( Reynolds and Lewis, 2017). Sharing ideas therefore supports the development of effective bioinformatics analysis. By offering an approach for researchers to share and discuss the methods and decisions involved in their analysis, the Thinking Process Template also promotes a deeper level of transparency in bioinformatics analysis. This includes not only the sharing of positive results, but also the sharing of negative or null results. In many cases, null results can be just as important to science, as they provide valuable information about what does not work and can help the broader community to avoid repeating failed experiments or approaches. However, the current scientific field leans more towards the reporting of positive results only. We see the Thinking Process Template to be a tool that can support the sharing of both positive and negative results by providing a structured framework for documenting the decisions and findings in various steps of the analysis. The document can later be shared with the community to increase the transparency of the work.

A distinct and complementary component to the Thinking Process Template is related to the ease for researchers to reproduce open data analyses on their local computer systems. Robustness of computational tools is an enduring issue in various analytically-driven fields and challenges with reproducing data analytics is often due to the difference in software versioning and the large variety of operating systems. To address these issues, the R programming community has developed tools such as BiocManager and Renv to help with the installation and documentation of R package dependencies. The use of containers such as Docker allows for the creation of fully reproducible software and analytical environments that can be easily shared and run on different operating systems. In the case of SCDNEY, we have taken steps to improve the robustness of the tool. The scdney wrapper package ( https://github.com/SydneyBioX/scdney) and its individual packages are incorporated into controlled repositories such as Github and Bioconductor. In addition, scdney is provided as a Docker container which contains all the necessary dependencies for installation, making it easy for researchers to install and use scdney on their local systems.

## Conclusion

In conclusion, the advancement of computational methodologies for integrative analysis of single-cell omics data is transforming molecular biology at an unprecedented scale and speed. Here we introduce the design Thinking Process Template that structures analytical decision making together with scdney, a wrapper package with a collection of packages presented in the context of several data stories. By establishing scdney as a collection of living workshops, we highlight the current solutions in generating novel biological insights. By emphasising the Thinking Process Template and the critical thinking process behind in our workshops, we aim to empower users to more effectively and confidently use scdney to gain insights from their single-cell data. Finally, we discuss various key aspects such as reproducibility, replicability, and usability of the computational tools. We hope scdney serves as a foundation for future development and application of computational methods for integrative analysis of and biological discovery from single-cell omics data.

## Author contribution

JY, SG conceived, designed and funded the study. HK completed the analysis and design of data story 1 with feedback from YL, PY. YC and AT completed the analysis and design of data story 2 with guidance from JY, and SG. The implementation and construction of the R package for the case study were done jointly between YC and AT. NR tested all R packages; MT and YL develop the graphics with feedback from JY, SG and EP. The development of the designed Thinking Process Template was done jointly by all authors and all authors wrote, reviewed and approved the manuscript.

## Data Availability

NCBI Gene Expression Omnibus (GEO): Transcriptome analysis of single cells from the developing mouse dentate gyrus. Accession number, GSE104323, https://identifiers.org/ncbigene:104323. European Genome-phenome Archive (EGA): ScRNA-seq of PBMC and whole blood samples reveals a dysregulated myeloid cell compartment in severe COVID-19. Access number EGAS00001004571, https://ega-archive.org/studies/EGAS00001004571.

## Software availability

Source code available from: https://github.com/SydneyBioX/scdneyDiseasePrediction/tree/v1.0.0.

Archived source code at time of publication: https://doi.org/10.5281/zenodo.7582777 ( Cao and Tran, 2023).

License: MIT.

Source code available from: https://github.com/SydneyBioX/scdneyAdvancedPhenotyping/tree/v1.0.0.

Archived source code at time of publication: https://doi.org/10.5281/zenodo.7582775 ( Lin, Kim and Chen, 2023).

License: MIT.
