# Supplementary material for: Thinking process templates for constructing data stories with SCDNEY
Source: F1000Res. 2023 Dec 15;12:261. Originally published 2023 Mar 10. [Version 2] doi: 10.12688/f1000research.130623.2 (PMC10905113; doi:10.12688/f1000research.130623.2)
Supplement: Peer review history

# Reviewer report: Reviewer response for version 2

**DOI:** 10.5256/f1000research.159911.r230731

**Published:** 2024-02-15

**Authors:** Kelly Street

**Affiliations:** 1 Department of Population and Public Health Sciences, Keck School of Medicine, University of Southern California, Los Angeles, California, USA

I appreciate the authors' thoughtful responses to my previous comments and I find the updated article clear and convincing. I have no further major comments and I am honored to have been included in this work!

 Following up on a couple (very) minor points:

 7.1. It doesn't look like this change was made in the updated version.

 7.2. The updated version still seems weird to me (I don't think it makes sense to "identify" pieces into a whole). I would rephrase with something like "Workflows can help users identify a set of seemingly disjoint methods and combine them into a unified and coherent process."

Is the work clearly and accurately presented and does it cite the current literature?

Partly

If applicable, is the statistical analysis and its interpretation appropriate?

Yes

Are all the source data underlying the results available to ensure full reproducibility?

Yes

Is the study design appropriate and is the work technically sound?

Yes

Are the conclusions drawn adequately supported by the results?

Yes

Are sufficient details of methods and analysis provided to allow replication by others?

Yes

Reviewer Expertise:

Single-cell transcriptomics, computational biology software development

I confirm that I have read this submission and believe that I have an appropriate level of expertise to confirm that it is of an acceptable scientific standard.

# Reviewer report: Reviewer response for version 1

**DOI:** 10.5256/f1000research.143393.r173874

**Published:** 2023-06-15

**Authors:** Kelly Street

**Affiliations:** 1 Department of Population and Public Health Sciences, Keck School of Medicine, University of Southern California, Los Angeles, California, USA

The authors present a Thinking Process Template for the analysis of complex datasets. This template encourages researchers to think critically about each step in their analysis and to be more transparent in the reporting of their results through the use of data stories that acknowledge the subjective decisions that had to be made along the way.

 The thinking process template is demonstrated through two example analyses of single-cell RNAseq data with methods from the scdney package. These examples are highly relevant, as a typical analysis in this field generally involves multiple interconnected steps with the choices made at each step having (potentially major) downstream consequences. These example data stories show what additional questions the researchers asked at each step of the analysis in order to establish confidence in their results to that point. These questions and the associated supplemental analyses are generally not included in the final products of most research projects (papers, software, etc.), but are of critical importance to the validity of the results.

 I strongly agree with the authors' call for greater transparency in method selection and the educational advantages of training analysts to think critically about their pipelines. While I found some of the concepts presented in this manuscript confusing, I think the overall message is both timely and critically important.

1. The biggest source of confusion for me was the notion of the Thinking Process Template. The authors claim that this template will "formalize the thought process an analyst should undertake to ensure robust analysis that is tailored to their data," which is quite a lofty goal. This template is referenced throughout and demonstrated by the two example analyses, but I was never clear on its definition or practical use (Figures 3 and 5 and Boxes 1 and 2 all seem more useful and generalizable than Figures 1 and 2). As such, I failed to see the connection between the different Results subsections. I think the paper could be improved by providing a more concise definition of the Thinking Process Template. Alternatively, I think removing/de-emphasizing these sections and focusing on advocating for the use of data stories (as demonstrated), would make for a more effective message, as I'm not sure what is gained from the additional terminology.
2. On a related note, the title mentions "Thinking process templates" (plural, not capitalized), whereas the text itself often refers to a Thinking Process Template (singular, capitalized). Given my confusion related to point (1), I found myself wondering if the "templates" actually referred to the example analyses, or if it was indeed supposed to be something more general.
3. It took me a little while to find the workshop materials mentioned, particularly for the second data story, which is not listed on the scdney website (at least, not the one I found: https://sydneybiox.github.io/scdney/ ). Since these materials are central to the results, it would be helpful to include direct links to them in the text, if possible, or otherwise in the Data/Software Availability sections (I'm referring to https://sydneybiox.github.io/scdneyAdvancedPhenotyping/articles/advanced_phenotyping.html and https://sydneybiox.github.io/scdneyDiseasePrediction/articles/disease_outcome_classification_schulte.html , and apologize if these are not the intended definitive versions of the data stories).
**Minor comments**
4. The full data stories make use of several methods that are not mentioned or cited, but perhaps could be. I noted Monocle 3, tradeSeq, velocyto, clusterProfiler, CellChat, and CYTOTRACE for the first data story and Seurat for the second.
5. Figure 4B could use more explanation. The top five marker genes seem to do a much better job of identifying the original set of "ImmGranule2" cells than the re-classified set. Why do these markers become less informative in the re-classified data? Wouldn't that indicate that the original labels were more biologically meaningful?
6. I think the phrase "through visuals, we enlighten the data" only works if you use the archaic definition of "enlighten". "Illuminate" might be a better choice.
7. The following sentences/phrases could stand to be re-written for clarity:
  1. Introduction: "...how 'robust' a data analysis should be conducted"
  2. Introduction: "...help users identify a set of seemingly disparate methods into a cohesive whole"
  3. Methods: "not from previous studies nor have them been published somewhere else"
  4. Figure 1: "Critical thinking questions to what we have to make from the Data, drive our decision with the Narrative and enlighten with the Visuals."
  5. Results: "...creates multi-view feature representation on patient level..."
  6. Results: "The scdney workflow start(s) with data..."
  7. Results: "...gene sets that distinguish hippocampal development in mice from scRNA-seq data..." (sounds like you are contrasting "hippocampal development" and "scRNA-seq")
  8. Conclusion: "...scdney, a collection of wrapper packages..." (scdney is the wrapper package)
8. The bulleted list of methods in the scdney package should be consistently formatted (ie. should all start with a lower case "a" or "an").

Is the work clearly and accurately presented and does it cite the current literature?

Partly

If applicable, is the statistical analysis and its interpretation appropriate?

Yes

Are all the source data underlying the results available to ensure full reproducibility?

Yes

Is the study design appropriate and is the work technically sound?

Yes

Are the conclusions drawn adequately supported by the results?

Yes

Are sufficient details of methods and analysis provided to allow replication by others?

Yes

Reviewer Expertise:

Single-cell transcriptomics, computational biology software development

I confirm that I have read this submission and believe that I have an appropriate level of expertise to confirm that it is of an acceptable scientific standard, however I have significant reservations, as outlined above.

# Response

**Published:** 2023-12-07

**Authors:** Yue Cao

**Affiliations:** The University of Sydney, Australia

Response:

 We thank the reviewer for the support in the transparency of method selection. Below we provide point-to-point response.  

1. The biggest source of confusion for me was the notion of the Thinking Process Template. The authors claim that this template will "formalize the thought process an analyst should undertake to ensure robust analysis that is tailored to their data," which is quite a lofty goal. This template is referenced throughout and demonstrated by the two example analyses, but I was never clear on its definition or practical use (Figures 3 and 5 and Boxes 1 and 2 all seem more useful and generalizable than Figures 1 and 2). As such, I failed to see the connection between the different Results subsections. I think the paper could be improved by providing a more concise definition of the Thinking Process Template. Alternatively, I think removing/de-emphasizing these sections and focusing on advocating for the use of data stories (as demonstrated), would make for a more effective message, as I'm not sure what is gained from the additional terminology.
  Response:
  We appreciate the reviewer’s perspective and would like to provide some clarification on the Thinking Process Template. Thinking Process Template is a general term that refers to the data analysis procedure from data input to final analysis outcome that involves critical thinking processes and goes beyond directapplication of tools. It is distinct to data stories which are the products of data analysis efforts. We have now clarified the terminology in the introduction section: 
*“We use Thinking Process Template as a general term to refer to the data analysis procedure from data input to final analysis outcome that involves critical thinking processes and goes beyond the simple application of tools or the products of data analysis.”*
2. On a related note, the title mentions "Thinking process templates" (plural, not capitalized), whereas the text itself often refers to a Thinking Process Template (singular, capitalized). Given my confusion related to point (1), I found myself wondering if the "templates" actually referred to the example analyses, or if it was indeed supposed to be something more general.
  Response:
  We refer to the “Thinking Process Template” as the general term that encapsulates the critical thinking process rather than specific templates. In single-cell, the analysis that researchers perform are diverse and there is no Thinking Process Template applicable to all tasks. Therefore, we provided two examples in Figures 3 and 5 and Box 1 and 2 to illustrate two examples of Thinking Process Templates in the context of two data stories: 1) the identification of gene pairs and 2) patient prediction.
3. It took me a little while to find the workshop materials mentioned, particularly for the second data story, which is not listed on the scdney website (at least, not the one I found: https://sydneybiox.github.io/scdney/ ). Since these materials are central to the results, it would be helpful to include direct links to them in the text, if possible, or otherwise in the Data/Software Availability sections (I'm referring to https://sydneybiox.github.io/scdneyAdvancedPhenotyping/articles/advanced_phenotyping.html and https://sydneybiox.github.io/scdneyDiseasePrediction/articles/disease_outcome_classification_schulte.html , and apologize if these are not the intended definitive versions of the data stories).
  Response:
  We thank the reviewer for this suggestion and have updated the https://sydneybiox.github.io/scdney/workshops.html to include the second data story.

Minor comments 

1. The full data stories make use of several methods that are not mentioned or cited, but perhaps could be. I noted Monocle 3, tradeSeq, velocyto, clusterProfiler, CellChat, and CYTOTRACE for the first data story and Seurat for the second.
  Response:
  We have added the relevant packages and references:
*“as well as asking which genes are differentially expressed across a pseudotime using tradeSeq (Van den Berge et al. 2020) and performing functional annotation of these gene sets through clusterProfiler (Yu et al. 2012). Together, these analyses demonstrate that the final trajectories are in line with our expectations and provide more confidence in the new biological insights extracted from these trajectories. The story includes other downstream analysis of the data such as cell-cell communication using CellChat (Jin et al. 2021) and RNA velocity analysis using scVelo (Bergen et al. 2020), which the users can perform to further explore their data.”*
2. Figure 4B could use more explanation. The top five marker genes seem to do a much better job of identifying the original set of "ImmGranule2" cells than the re-classified set. Why do these markers become less informative in the re-classified data? Wouldn't that indicate that the original labels were more biologically meaningful?
  Response:
  We apologise for the confusion. We have corrected the mislabel in the legend. The visualisations show that the cell-type specific markers show a better distribution in the newly annotated cell type group (blue, re-classified) than in the original grouping (red, original). A higher expression of the top marker genes suggests that new groupings now better represent the cell-type profile of ImmGranule2.
3. I think the phrase "through visuals, we enlighten the data" only works if you use the archaic definition of "enlighten". "Illuminate" might be a better choice.
  Response:
  We have now replaced the word “enlighten” with “illuminate”, including the occurrence in Figure 1.
4. The following sentences/phrases could stand to be re-written for clarity:
  1. Introduction: "...how 'robust' a data analysis should be conducted"
    Response:
    We have rephrased this into: *“Hence, this can create challenges when it comes to deciding how rigorous a data analysis is.”. *
  2. Introduction: "...help users identify a set of seemingly disparate methods into a cohesive whole"
    Response:
    We have rephrased this into: *“Workflows can help users identify a set of seemingly disjoint methods into a unified and coherent process.”*
  3. Methods: "not from previous studies nor have them been published somewhere else"
    We have rephrased this into: *“These two data stories and the accompanying workshops were not derived from previous studies nor have they been published elsewhere.”. *
  4. Figure 1: "Critical thinking questions to what we have to make from the Data, drive our decision with the Narrative and enlighten with the Visuals."
    Response:
    We have rephrased this into *“Critical thinking questions from the data drive our decision with the Narrative and illuminate with the Visuals.“ *
  5. Results: "...creates multi-view feature representation on patient level..."
    Response:
    This is now corrected.
  6. Results: "The scdney workflow start(s) with data..."
    Response:
    This is now corrected. 
  7. Results: "...gene sets that distinguish hippocampal development in mice from scRNA-seq data..." (sounds like you are contrasting "hippocampal development" and "scRNA-seq")
    Response:
    We have changed the word “from” to “using” to avoid the confusion. 
  8. Conclusion: "...scdney, a collection of wrapper packages..." (scdney is the wrapper package)
    Response:
    We have corrected this sentence.
5. The bulleted list of methods in the scdney package should be consistently formatted (ie. should all start with a lower case "a" or "an").
  Response:
  This is now fixed. 

# Reviewer report: Reviewer response for version 1

**DOI:** 10.5256/f1000research.143393.r170725

**Published:** 2023-05-23

**Authors:** Jun Li

**Affiliations:** 1 Department of Human Genetics, University of Michigan, Ann Arbor, Michigan, USA

This article described an important effort to improve the teaching of data science skills through "thinking process templates". In the past, static knowledge was taught in didactic lectures, whereas data analysis workflow was taught in step-by-step tutorials. What remains lacking is to treat the analysis of each dataset as a unique journey, containing decision points that require context-specific diagnosis and judgement. This article focused on single-cell data analysis in the scdney system, which is a collection of related R packages performing data integration, cell type annotation, statistical modeling such as differential expression analysis and, importantly, "constructing data stories".

 Such an effort is much needed, as most of the time the tutorials for popular analysis workflows were presented as a single path through the data, without highlighting key forking points, measures of confidence, and reasons to select certain algorithms for normalization, projection, or visualization. The article stated this problem very well, and sought to design the thinking template to uncover "hidden pitfalls and assumptions".

 It also drew attention to the importance of the "interdependence" of data analysis steps (such as imputation-then-normalization versus normalization-then-imputation), as the choices "have cascading impacts on the downstream analysis".

 The main part of the article described two use cases, using different datasets for different end goals. They illustrate how thinking process needs to be customized for the situation, even though the basic codes and the statistical principles are similar. The single most important value of such an exercise is to advocate for a new way of combining scholarship with pedagogy, in which the analysts strive to provide "a structured framework for documenting the decisions and findings in various steps of the analysis".  As such, the emphasis is to go beyond the practice of recording the workflow, by being responsible for sharing the reasons, alternative decisions, and inevitable compromises as one brings a story out of a data object.  

 In terms of improvement, my main suggestion is to highlight one or two examples where the standard workflow would proceed unwittingly into the wrong decision (such as using the default choice of normalization method or embedding parameters), but a more circumspect, thinking-along-the-way approach would have produced relevant diagnostics and turned the analysis unto another path. Similarly, it would be useful to provide an example where a decision depends on the earlier choices, or when the same dataset needs different treatments for different goals.

 Very minor issues:

1. A typo in page 4 "reocrded".
2. Please concisely explain "differential stable genes" and "higher-order changes", in page 5.
3. In the paragraph before "Conclusions", in the sentence "To address these issues,…" there is probably a misplaced comma.
4. In Conclusion, the sentence "Together with scdney,…" needs fixing.

Is the work clearly and accurately presented and does it cite the current literature?

Yes

If applicable, is the statistical analysis and its interpretation appropriate?

Yes

Are all the source data underlying the results available to ensure full reproducibility?

Yes

Is the study design appropriate and is the work technically sound?

Yes

Are the conclusions drawn adequately supported by the results?

Yes

Are sufficient details of methods and analysis provided to allow replication by others?

Partly

Reviewer Expertise:

Single-cell data analysis, data science education, data processing and modeling

I confirm that I have read this submission and believe that I have an appropriate level of expertise to confirm that it is of an acceptable scientific standard.

# Response

**Published:** 2023-12-07

**Authors:** Yue Cao

**Affiliations:** The University of Sydney, Australia

We thank the reviewer for supporting our teaching philosophy on critical thinking. We have now re-written the section on the second data story to highlight examples where critical thinking is needed for diagnosis.

 The modified section is copied below:

*“Once the final models are obtained, we ask questions on the robustness of the models. One approach on this involves assessing the generalisability of the model on an independent dataset. We examined the performance of the 13 models on a different COVID-19 dataset obtained from the Wilk study that also contain mild and severe patients (Wilk et al., 2020). We found while the 13 models have close balanced accuracy between 0.75 and 0.88 on the Schulte-Schrepping dataset, their performance varied greatly in the Wilk dataset and ranged between 0.49 to 0.78 (Figure 6A, B). It is noteworthy that models built from feature types such as “gene proportion cell type” that have high accuracy do not necessarily maintain good performance on the Wilk dataset. On the other hand, the feature type “CCI” achieved an accuracy of over 0.75 in both datasets, indicating potential for further examination. Finally, to extract biological insights from the fitted models, we guide users to interpret the fitted models to identify important features and reflect on whether the features make sense. Here, it is important not to select the top features based on a single model, but to ask about the stability of these top features. To illustrate this idea, we examined all features that appeared at least once as top 10 features in the cross-validated models. Figure 6C highlights that while the majority of the features were consistently ranked as top features across all models, a proportion of features were ranked in the hundreds and thousands position in some models. These two scenarios illustrate the importance of critical thinking to avoid heading down a wrong decision path.”*

 Below we provide point-to-point response on the minor issues. 

 Very minor issues:

1. A typo in page 4 "reocrded".
  Response:
  This is now fixed.  
2. Please concisely explain "differential stable genes" and "higher-order changes", in page 5.
  Response:
  We have added a concise description to each of the terms: “ *a novel method for identifying differential stable genes, that is, genes that are stably expressed in one cell type relative to other cell types” and “By higher-order changes, we refer to higher order interactions such as variation and coexpression that are beyond changes in mean expression”. *
3. In the paragraph before "Conclusions", in the sentence "To address these issues,…" there is probably a misplaced comma.
  Response:
  We have rephrased this sentence into: “ *To address these issues, the R programming community has developed tools such as BiocManager and Renv to help with the installation and documentation of R package dependencies.”*
4. In Conclusion, the sentence "Together with scdney,…" needs fixing.
  Response:
  We have rephrased the sentence into: “ *Here we introduce the design thinking process template that structures analytical decision making together with scdney, a wrapper package with a collection of packages presented in the context of several data stories.”*
